# Guidelines for Scrutiny of Death Associated With Surgery and Anesthesia

**DOI:** 10.7759/cureus.70841

**Published:** 2024-10-04

**Authors:** Heba E Mostafa, Eman A Alaa El-Din, Abd Almonem A Albaz, Dena M Abdel Moawed

**Affiliations:** 1 Forensic Medicine, Faculty of Medicine, Al-Rayan Colleges, Al-Madinah Al-Munawwarah, SAU; 2 Forensic Medicine and Clinical Toxicology, Faculty of Medicine, Zagazig University, Zagazig, EGY; 3 General Administration of Criminal Evidence, Ministry of Interior, Kuwait, KWT; 4 Forensic Medicine and Clinical Toxicology, Badr University, Cairo, EGY

**Keywords:** anesthetic mortality, autopsy, intraoperative deaths, medico-legal, postmortem examination, postoperative deaths

## Abstract

The death of a patient in the operating room frequently causes great distress to the patient's family, and the surgical team members who performed the procedure typically feel uneasy about it afterward. Anesthetic death is characterized as a death that happens 24 hours after anesthesia is administered and is caused by anesthetic-related factors. However, death can come much later because of its complications. This review thoroughly explains mortality resulting from surgery and anesthesia, including autopsy reports, investigative data, analytical techniques, and conclusions. Following surgery and anesthesia-related death, in case of death after surgery, an autopsy determines the cause of death and if the procedure had any impact on it. Individuals who pass away during or after surgery may do so for a wide range of reasons, such as a generally natural condition, an early or late surgical complication, an anesthetic problem, or an error during the operation or anesthesia. The pathologist should take the results of the examination into account while looking into an anesthetic death. In the majority of anesthesia-related deaths, autopsies reveal little diagnostic information with the absence of an underlying cause of death. The analyses help determine and estimate the dosage of medication given, as well as the amount of anesthetic agent overdose provided before death. The best consensus opinion to provide the investigative authorities and courts of law for the cause of death investigation may come from a discussion amongst forensic pathologists, surgeons, and anesthetists. In conclusion, the family and friends of the deceased are greatly affected by an unexpected or unexplained death, and the organizations entrusted with determining the cause of death bear a great deal of responsibility. Science and technology are becoming more and more important in death investigations. Following precise and well-founded procedures is one of the science's defining characteristics.

## Introduction and background

The phrase "perioperative death or death associated with surgery and anesthesia" is vague, and because physicians at all levels are uninformed of their legal obligations, deaths resulting from invasive diagnostic procedures and anesthesia are frequently not reported to the proper authority [1]. Anesthetic death is defined as death occurring within 24 hours of administration of anesthesia due to causes related to anesthesia. However, death may occur even afterward due to its complications [2]. Surgical death is defined as individuals undergoing surgical interventions who may die during the intervention (intraoperative deaths) or in the postoperative period (following transfer from the post-anesthesia care unit to an intensive care unit or general ward). Deaths relating to the operation may occur as a result of issues quite some time after surgery. Death may be due to the disease for which the operation was performed, a complication of the operation and its anesthetic, or an unrelated factor [3].

Four subgroups can be identified based on the cause of death for these cases [4], namely: 1) Those directly brought on by the illness or injury that the invasive operation or anesthesia was used to treat; 2) Those brought on by an illness or problem other than the one for which the surgery was carried out; 3) Those brought on by a mistake or complication that arose during the invasive operation; 4) Those brought on by a mistake or complication that arose while performing an aesthetic operation.

The guidelines are methodically formulated statements grounded in the most reliable data to aid and encourage the choices made by practitioners. The guidelines function as instruments for executing and propagating optimal methodologies. The Royal College of Pathologists gave the guidelines "Guidelines on autopsy practice: Postoperative deaths" to cover most typical situations. Nevertheless, not every pathology specimen or clinical situation can be considered by those standards. It may, therefore, be necessary to occasionally deviate from this guideline's recommended approach to document a case in a manner most advantageous to forensic doctors, authorities, and the family of the dead [3]. They adhere to standard postmortem examination criteria when it comes to analyzing deaths involving surgery and anesthesia, although some of them specifically address such deaths (Table 1).

**Table 1:** Guidelines for investigating deaths related to surgery and anesthesia ACP: Advance Care Planning

	Guidelines	References
1	ACP Best Practice no.155: Pathological investigation of deaths following surgery, anesthesia, and medical procedures	[5]
2	Guidance for pathologists conducting postmortem examinations on individuals with implanted electronic medical devices	[6]
3	Guidelines on autopsy practice: Sudden death with likely cardiac pathology	[7]
4	Guidelines on autopsy practice: Autopsy when drug overdose or poisoning may be involved	[8]
5	National Confidential Enquiry into Patient Outcome and Death (NCEPOD)	[9]
6	Guidelines on autopsy practice: Postoperative deaths	[10]
7	Guidelines on autopsy practice: Autopsy examination following bariatric surgery	[11]
8	Guidance for doctors completing medical certificates of cause of death in England and Wales	[12]
9	Guidelines for best practice: Principles for radiographers and imaging practitioners providing forensic imaging services	[13]

Introduction

Postoperative fatalities, which happen during or within 30 days of a surgical procedure, are common. Forensic pathologists may encounter challenging autopsies during the investigation of these fatalities. The patient often has a long clinical history; their anatomy is deformed by disease and therapy, and many clinical questions need to be answered [14]. The lack of an agreed-upon definition of anesthesia and perioperative-related deaths is a serious difficulty in the forensic area, and the terms "operative deaths" and "anesthetic deaths" are sometimes used interchangeably in the medico-legal novel. From the perspective of forensic pathology, at least, these incidents involving perioperative mortality permit a fine differentiation between natural and unnatural death. In this sector, iatrogenic deaths can be divided into multiple groups based on whether they resulted from the surgical process itself (e.g., perioperative cardiac and cerebrovascular events) or from the adverse circumstances of patients' prior conditions [15]. When a patient is moved from the post-anesthesia care unit to an intensive care unit, they may pass away during the procedure (intraoperative fatalities) or after (postoperative deaths) [16]. Complications that develop after the procedure may be the cause of death due to the procedure. Death may result from an unrelated source, a consequence of the operation and anesthesia, or the disease for which the procedure was conducted. The next autopsy will differ in complexity; therefore, a comprehensive, methodical approach is necessary. With the coroner's approval, it could be advantageous to have the operational surgeon and other physicians engaged in the patient's care attend the autopsy, even though the pathologist performing the autopsy should reach their conclusions [10]. 

A vast and growing list of disorders can be surgically treated, palliated, or cured, making surgery a vital component of health care systems. Three hundred thirteen million surgical procedures are performed worldwide every year. In low- and middle-income nations, emergency patients account for 60% of surgical operations performed annually, yet an additional 143 million surgical procedures are required to prevent disability and save lives. Surgical operations are not without risk, and there is always a risk of death owing to the process or the anesthesia used during the treatment, as well as postoperative complications when considering the patient's condition at the time of surgery [17]. Ischemic heart disease and stroke are thought to be the two leading causes of death worldwide, with postoperative deaths coming in third. Half of postoperative fatalities occur in low- and middle-income countries, even though 4.2 million people die within 30 days of surgery each year, and postoperative fatalities account for 7.7% of all deaths worldwide [18]. The postoperative mortality rate for elective procedures in Africa is twice as high as the global norm, according to the Lancet Commission on Global Surgery (LCoGS) [19]. Usually, with the aid of an autopsy, the proper medico-legal authority (coroner or procurator fiscal) will look into intra- and postoperative deaths. It is commonly known that a well-executed autopsy will identify a huge number of cases in which death could have been prevented if the right diagnosis had been made earlier in life [20]. An autopsy can help establish or disprove the clinical diagnosis of the cause of death in cases where malpractice is suspected. It can also reveal pathologies and complications that were not suspected during the patient's lifetime but may have affected the cause of death and management choices [21]. One would expect the autopsy to be carried out to a high standard given the frequency of these deaths each year and the fact that these investigations are of interest to the bereaved relatives of the deceased as well as to the surgeons, anesthesiologists, and other staff members who cared for the patient during his or her life [22]. Excluding reports involving suspected homicide, the National Confidential Enquiry into Patient Outcome and Death (NCEPOD) evaluated the quality of coronial autopsy reports in 2006 and found that over 25% of them were inadequate or unsatisfactory. The Royal College of Pathologists recently released guidelines to raise the standard of autopsies performed during and after surgery [9].

Investigating the cause of death prerequisites broad discussion between forensic pathologists, surgeons, and anesthetists in order to arrive at the best consensus of opinion to offer the investigating authority and courts of law. They must come out with protocols to be followed in different clinical situations. This review aims to demonstrate the guidelines for investigating deaths related to surgery and anesthesia, with a focus on deaths after bariatric surgery, individuals with medical devices, drug overdoses, and anesthesia-related deaths. The significance of the autopsy report and the interpretation of the cause of death are also considered.

## Review

Postmortem examination (autopsy)

Objectives of the Autopsy

An autopsy is performed when a patient passes away following surgery in order to know the cause of death and the influence, if any, that the procedure may have had on it. Surgical or postoperative deaths can occur for several reasons. The investigator's role is to answer the following questions [23]: Was the medical procedure responsible for or contributed to the death, and if so, how? Did the death result from a natural disease or other preexisting conditions, and if so, how? Did the patient provide a correct pre-surgical assessment, especially regarding the nature and severity of the extent to which the procedure was being performed and the operating risk? Was the quality of medical care before, during, and following the procedure sufficient? The pathologist or investigator will respond to the preceding questions based on the operation's nature, the patient's age, comorbidities present or absent, and the time between surgery and death.

Preparation for Autopsy and Postmortem Examination

The pathologist should be able to identify the main topics for study after carefully reviewing the case; if there is any ambiguity, further evaluation and consultation may be necessary [5, 24]. The investigation of surgical and anesthetic-related deaths brings up certain difficulties. The autopsy may be technically challenging, and there may be few or no gross morphological findings due to the surgical procedure. Normal anatomy can be distorted by exudates, infections, adhesions, hemorrhage, and edema; therefore, it is critical to understand all previous surgeries. It is important to consider keeping blood and bodily fluid samples after death. While biochemistry and hematology laboratories may discard samples immediately, blood transfusion and serology laboratories normally keep samples for days or weeks. These could be useful in evaluating creatine phosphokinase activity in malignant hyperthermia, confirming disseminated intravascular coagulation or identifying problems with blood transfusion.

The reporting pathologist should also go over both recent and older histology specimens. Requesting laboratory test data obtained during life but unavailable at the time of death can be helpful. Although interpretation may be difficult, blood cultures and cardiac enzyme levels may provide useful information regarding the cause of death. Several medical devices, such as subcutaneous pacemakers, nasogastric tubes, urine catheters, wound drains, chest drains, and metal or plastic prostheses, may have been implanted in the patient. No article must be removed before the autopsy, so pathologists must make advance arrangements with wards, intensive care units, imaging departments, and operating rooms to avoid any of these from being removed. It may be necessary to confirm the location and patency of all these devices during necropsy. All anesthetic machines must use the same technique. When there are many tubes around, things can go wrong. Mistakes may occur, for instance, if a prescription drug is injected into the wrong tube, like a central venous line filled with stomach medication.

Timing of the Autopsy

The role of infections in the cause of death is a prominent concern in autopsies looking into postoperative deaths. Within an hour of death, postmortem bacterial translocation and putrefactive decomposition start, which ultimately results in corpse breakdown. Thus, the necropsy can be started following a comprehensive examination of the patient's antemortem history, including pertinent serum investigations and positive and negative cultures. The proper personal protection equipment must be used to prevent catching an infectious disease or transferring it to others [25]. It is often desirable to start the autopsy as soon as possible.

Important Information Before the Autopsy

Ideally, autopsies for perioperative deaths ought to be carried out only following a review of the clinical history. This aids the autopsy pathologist in organizing the autopsy examination and deciding which questions to try to answer during the autopsy. The surgical, anesthetic, and nursing teams should be consulted for any concerns. According to Jenkins et al., it is important to be particularly aware of the following details when examining the clinical history [22]: pre-admission and admission clerking (prior diagnosis, medication history, and allergy history); Obtaining consent forms prior to the procedure; operation notes; drug identification cards; postoperative treatment; any results from laboratory chemistry, hematology, microbiology, or antemortem histology (they help provide a complete picture and may avoid additional tests).

Autopsy Dissection (Postmortem Examination)

External examination: After a perioperative death, an external examination usually determines the route to internal structures and any devices present. Consequently, a complete physical examination is necessary to detect any surgical incisions or scars, cannulae, and previous sites of line, catheter, drain, fixator, etc. If relevant, these should be noted along with their size and any indications of infection, poor healing, or other illnesses [20, 26].

Saukko et al. recommended documenting all anesthesia and surgical equipment, preferably on a body chart [27]. Similarly, precise anatomical descriptions and dimensions should be recorded for all surgical incisions and scars, including those from ileostomies and colostomies. Documentation is necessary to determine the kind and state of any current dentition and the extent of cyanosis, jaundice, and edema. Skin rashes are common and can be severe, especially when immunosuppression, septicemia, internal malignancies, and drug reactions are present. All bruise regions and locations related to probable external device insertions should be noted. Other reasons for bruising, such as anticoagulant therapy or thrombocytopenia, should be thoroughly investigated.

Nutritional status, edema, jaundice, tissue swelling, and ulceration should all be considered when assessing general body health (often decubitus). Photography can reveal signs of a sepsis-related condition, such as marbling and disseminated intravascular coagulation. If sepsis is suspected, relevant microbiology samples should be collected before opening the body [28].

Internal examination (autopsy procedures): A standard Y-shaped incision should be made to open the body methodically. Digital autopsy and digital photography are combined in postmortem computed tomography (PMCT). A methodical approach to evisceration is necessary; the body should be opened using a standard Y-shaped incision unless the scope of the interior inspection will be restricted after PMCT.

The importance of digital autopsies and photos: According to Vester et al., PMCT is a growing trend in digital autopsies [29]. While it is widely utilized worldwide, it is not yet available in every center. It has been demonstrated that PMCT reduces the need for intrusive autopsy examinations in patients who passed away naturally. It can identify mortality connected to infections when used with minimally invasive sampling. It has been suggested in recent years to employ PMCT as a non-invasive, quick, inexpensive, and widely accessible method of figuring out the cause of death. This could enhance mortality statistics, healthcare quality management, preventative programs, and perhaps even the identification of genetic illnesses that have already been discovered. When an autopsy is not a possibility due to trauma, PMCT has shown to be a useful substitute. In addition to helping with cause of death diagnosis, PMCT can help identify underlying disorders.

Incisions: The standards of a routine autopsy will usually apply to the dissection of the body; no equipment or prior surgical or medicinal intervention will be used. The type and placement of the primary incision will depend on each case's specifics and personal preference. It is customary to start incisions for the head and neck area behind each ear, proceed over the posterior-lateral aspect of the neck, and cross the clavicles over the outside third of the neck. Then, a curve connecting these two incisions is made, meeting above the sternum. This combination makes the neck structures visible and makes installing any indwelling devices precisely possible [30].

Usually, the midline anteriorly is where incisions are done in the chest and belly. To ensure proper placement assessment, carefully dissecting inserted devices is important. Air embolism, pneumothorax, and surgical emphysema must be ruled out at this early stage. All body organs and cavities are visible after the anterior rib cage is removed, and the skin is reflected before further dissection. All pus, hemorrhages, effusions, and other fluids should be documented and sampled. Some bleeding is normal at surgical sites, although more than 250 ml is uncommon [27].

A thorough autopsy necessitates knowledge of the previous surgical interaction since once lines and electrodes are cut, they can never be fully evaluated. Photographs taken during dissection often yield valuable information for evaluation later [31, 32].

According to Diac et al., in order to prevent contamination from external sources, blood and microbiological samples should be obtained before further dissection [33]. Getting blood cultures from the heart or spleen after the surface has been scorched to eliminate impurities is ideal. After cardiothoracic surgery, sepsis should be looked for in the thorax, subphrenic, and pelvic regions and surrounding the surgical site in cases where septicemia is known. It is important to rule out immunodeficiency, malnourishment, persistent drinking, and malignancy as probable causes of septicemia. To educate them and evaluate the case within the framework of the entire institution, hospital-acquired infections should be revised with the infection control unit. After abdominal surgery, the intestines should only be examined very carefully. Autolysis can complicate appearances, particularly in cases where anastomosis sites have recently undergone suture lines. During a necropsy, careless tissue handling could rupture important structures and impede an accurate assessment. Accurately detecting local postoperative problems can greatly benefit from the surgeon's presence. Surgical sites must always be inspected in situ before dissection; otherwise, artifactual abnormalities in organs and anastomoses may occur [34].

Organ removal (evisceration): As usual, the body cavities must be opened sequentially. Incisions made outside conventional protocols may be necessary to check for drainage lines and other devices in the subcutaneous and dermal tissues, depending on the situation [35]. Unconventional methods of opening the bodily cavities may also be used to check for implant-related issues. It is often better to assess the position and status of the device before the beginning of evisceration, like disembowelment, rather than after organs and tissues have been removed (e.g., bleeding, infection, misplacement). Photography of any pathology is helpful as the situation develops [9, 27].

Evisceration should, therefore, include [22]: i. accessing the internal organs; ii. physical examination of the contents of bodily cavities before organ removal; iii. observing the location of the device and whether local issues are present; iv. removal of devices such as nerve stimulators, pacemakers, and defibrillators. This might be simpler if radiology has established the devices' location and range. A thorough inspection of the tissue parenchyma may reveal lesions such as scarring and infection because the devices traverse the boundaries of skin and other tissue zones; v. checking of surgical anastomoses for dehiscence indications. It is crucial to avoid applying tension or traction to the anastomotic site when dissecting anastomotic sites, especially those in the intestine, as this could unintentionally rupture the tissues.; vi. Obtaining pictures of devices and organs in situ; vii. organ removal as usual and being cautious not to induce artifactual dehiscence or place tension on anastomoses.

Specific issues to be considered during postoperative death autopsy

Surgical Scars

Scars should always be mentioned in the report, even if only to state that there are or are not visible surgical scars. It is not necessary to categorize scars as tiny, medium, or large based on the anatomical location in which they are located. Scars can be raised, flat, curved, uneven, round, or linear, among other shapes. If there are several scars in a case of surgery-related death, it is helpful to have a distinct paragraph for scars. Scars should be thoroughly described, including the age and whether or not there are any indications of infection [34].

Organ Preservation

In most situations, entire organ preservation is unnecessary, particularly when imagery is available. According to the Guidelines on Autopsy Practice: Sudden Death Due to Cardiac Pathology, the heart may be preserved after difficult cardiac surgery for extensive examination by a cardiac pathologist [36].

Suspected Viral Infections

Virology samples do not have to be kept with every autopsy. Samples should be taken for virology whenever there is a possibility that a viral infection caused or contributed to the death. It is necessary to have pieces of brain tissue, myocardium, and solid lung tissue in addition to a nasal or bronchial swab. For analysis, save any pertinent fluid specimens, such as cerebrospinal (CSF) fluid. Caution must be exercised when dealing with suspected COVID-19 cases [37].

If an autopsy is performed on a suspected COVID-19 case, the following postmortem swab specimens should be collected and and tests should be performed for SARS-CoV-2 testing [38]: swab of the upper respiratory tract: nasopharyngeal swab; lower respiratory tract swab: swab from each lung. For testing for clinical diagnostic detection of SARS-CoV-2, reverse transcription polymerase chain reaction (RT-PCR) remains the "gold standard." In case of influenza virus and other respiratory pathogens, postmortem swab specimens should be collected. For additional postmortem microbiologic and infectious disease testing, autopsy tissues from the lung, upper airway, and other major organs that have been formalin-fixed paraffin-embedded (FFPE) (e.g., heart, liver, kidney) must be collected. In some cases, submitting FFPE autopsy tissues to the CDC for SARS-CoV-2 testing may be necessary.

Tissue Specimens for Histopathological Examination

Lucas et al. addressed how gross examination may miss infections, ischemia, and embolic events; as a result, it is usually advisable to have the major organs, generally the heart, lungs, liver, and kidneys, histopathologically examined. Although it may not always be necessary to sample these four primary tissues, it can yield valuable insights into the dying individual's overall pathophysiology and any possible contributing factors to their death, such as sepsis, disseminated neoplasia, or disseminated intravascular coagulation [39].

Heart samples can include one or more native/graft vascular segments. These segments can usually be placed in the same block, but thorough investigation often necessitates decalcification. One or two cardiac tissue blocks may be used for examination, but it may also include many samples from the tissues at the midventricular level and the cardiac conduction tissues (pacemaker cases) [3].

Tissue Sampling

Recommended tissue sampling according to Rous et al. [3] is described in Table 2.

**Table 2:** Recommended tissue sampling Source: [3]

Organ	Recommended sampling
Heart	Five blocks from a mid-horizontal slice: the anterior and posterior right ventricles and the four quadrants of the left ventricle. If stenosed, epicardial coronary arteries
Lungs	If a death has occurred following orthopedic surgery, one sample from each lower lobe is used for the frozen section, and Oil Red O staining is used to look for fat emboli.
Spleen	Whole organ
Liver	250 gm of tissue
Pancreas	Collect a sample if pancreatitis is suspected or if death occurs after pancreatic surgery.
Kidney	100 gm from both kidneys
Brain	100 gm of brain tissue
Bone	The lumbar vertebral bone is where surgery has been undertaken to treat osteoporotic fractures. This also allows CD68 assessment of marrow in cases of sepsis.
Other	Anastomotic sites, septic foci not already sampled, histopathological examination of any specimens removed at surgery

Toxicological Analysis

If the clinical history indicates that a drug overdose or metabolic disease (such as ketoacidosis) was the cause of death, samples ought to be gathered for toxicological examination. The pathologist should look into the availability of blood samples obtained during life, as these are frequently the most important for analysis. Blood, urine, vitreous humor, and stomach contents ought to be taken at the autopsy [15].

Toxicological analysis samples are always taken from a complete autopsy. Nonetheless, "needle autopsies," which are minimally invasive techniques for taking bodily fluid samples in place of a full-scale autopsy, have been used in some labs. Blood and urine from "needle autopsies" form the basis for 40%-60% of all toxicological investigations [40].

Microbiology Analysis

Before opening the body, blood samples for culture from the neck, veins, or the heart must be taken if the clinical history suggests sepsis as the cause of death. Extra samples may be necessary if the history and macroscopic findings indicate a specific infection focus. Bacterial samples for microscopy, culture, and sensitivity must be collected when there is a clear focus. If a patient's history suggests sepsis but there is no clear focus, the following samples mentioned in Table 3 must be collected [10].

**Table 3:** Recommended microbiological sampling Source: [39]

Tissue	Samples
Blood	0–20 ml from the neck veins or heart must be aspirated before opening the body
Lungs	One sample from each lower lobe.; each sample must be collected using new sterile instruments.
Spleen	One sample to be collected with sterile instruments when opening the abdomen
Bile	5–10 ml to be aspirated from the fundus of the gallbladder on opening the abdomen
Urine	5–10 ml to be aspirated from the fundus of the urinary bladder either before opening the body or upon opening the abdomen. The bladder is to be opened using sterile instruments and a bacteriological swab if the bladder is empty.
CSF	5–10 ml to be aspirated from the cisterna magna before opening the body
Others	Any apparent focus of infection is to be swabbed; The sites of occult sepsis – discitis, psoas abscess, and middle ears are to be examined

Autopsy Imaging

To determine the accurate cause of death and provide pre-dissection information, autopsy imaging is utilized. Postmortem imaging, which excludes pathological dissection, is simply imaging that is done after a person has passed away. Conversely, autopsy imaging offers imaging results that direct dissection and feedback autopsy imaging based on dissection results serves a supplementary function [41]. For many years, body imaging has been possible. Plain X-rays have traditionally been employed, although postmortem computed tomography and magnetic resonance imaging are now available to varying degrees. An external evaluation is recommended for autopsy cases requiring imaging assessments before proceeding to radiological review unless the mortuary scans all cases. This is especially useful for assessing bone structures and bodily compartments using transverse sections or reconstructed images after numerous traumas. Injecting dye into veins or hollow structures can help identify leaks, thrombosis, and blockages [42]. Current guidelines from the Royal College of Pathologists/Radiologists state that a comprehensive exterior examination of the body by a medical practitioner with the necessary training and credentials should be done before performing an imaging-based postmortem examination [43].

Guidelines for performing autopsy: special cases

Autopsy Examination After Bariatric Surgery Following Guidelines on Autopsy Practice (April 2019)

In such a case, the autopsy's role is to determine whether the bariatric surgical treatment caused the death, whether it was unrelated, whether it was natural, to assess the surgical anastomoses' integrity, and to find any comorbidities that might have aided in the patient's demise. Bariatric patients present a unique health and safety problem for the mortuary. Trays, trolleys, and tables must be able to support more weight, but refrigerated rooms must also be the proper size to accommodate a patient. The mortuary should ensure it has the tools needed to weigh and transport an increasing number of bariatric patients, and the scales must be calibrated regularly. If local storage is full or otherwise unavailable, contingency plans, such as service level agreements, should be in place to serve bariatric patients at other facilities [3]. Performing a bariatric postmortem requires substantial manual handling. A lateral movement is required to convey the corpse from the trolley to the postmortem table. Transferring 15-20 kg at waist level is advised by manual handling rules; this requires many workers to guarantee safety. The deceased is rolled so the pathologist can examine the back. Exercise caution to reduce the danger of musculoskeletal issues. Future mortuary designs could benefit from trays that lock onto tables to accommodate bariatric patients. Obese patients face technical challenges during evisceration due to increased subcutaneous fat, which can hinder skin reflection.

Additionally, anatomical pathology technicians and pathologists may struggle to visualize the blade during the procedure. Reducing neck extension makes removing the brain through a respectable incision more difficult. Excess soft tissue might cause the sutured skin incision to pull open, making body restoration more challenging. Local practice involves stitching the deep fascia before the skin to reduce tension on the suture line [11].

Postoperative Death When Drug Overdose or Poisoning Is Suspected

When drug overdose or poisoning may be present, an autopsy is performed [8]. The purpose of an autopsy is to determine whether a drug or toxin caused the postoperative death, whether it was due to another cause (such as positional asphyxia/pneumonia or a combination of both), what the pathological effects of drug use or misuse were, whether prior drug use caused any traumatic injuries, whether there was a preexisting illness that could have made a person more susceptible to the effects of a drug or toxin, whether the toxicity could have been treated to avoid death, and to gather suitable samples for toxicological analysis. Information needed before the autopsy is crucial since it will be included in the final report, along with relevant case history and information source data. It is impossible to overstate the value of a comprehensive history. The toxicology lab should receive all samples that have been gathered (Table 4).

**Table 4:** Types of samples for toxicological analysis with a special intimation Source: [8]

Samples		
Blood	In most postmortem cases, blood remains the most important specimen to analyze. At least 10 ml of peripheral blood (femoral or iliac) is suggested.	Caution: the gels used in many serum gel tubes may absorb drugs and thus affect the blood concentration.
Urine	At least 20 ml of urine should be collected in postmortem cases.	
Vitreous humor	Samples should be collected routinely in appropriate cases. At present, vitreous humor is used primarily to quantitate ethanol, urea, electrolytes, and beta-hydroxybutyrate.	All vitreous humor from both eyes should be collected; however, it can be collected into a single container. Following removal, the shape of the eyes can be restored by injecting water.
Gastric contents	The most important investigation is the observation of undigested pills and tablets. If these are present, they should be separated and placed into plastic pillboxes for analysis.	Stomach content is heterogeneous. If only an aliquot of stomach content is collected, the total volume/weight should be recorded.
Other samples		
Injection site (skin)	It may be useful in determining the type of substance that has been injected, such as insulin or heroin. Again, it is rarely required but needs to be considered. Always send a control site sample for comparison.	Excise a wide skin ellipse down to subcutaneous tissue to sample the injection site. Place the specimens in clean, labeled universal containers. If the specimen is for histology, add neutral buffered formalin. When fixed, examine and serially slice; if a tract is not identified, submit the entire specimen for histological examination. Otherwise, do not fix the specimen; instead, send the specimen immediately to the laboratory.
Lung tissues	Approximately 2 cm cubed, sealed in a glass airtight container or universally wrapped in parafilm, may be useful.	
Bile	It can be useful for screening (but not quantitation) if no other samples are available.	
Liver (deep within right lobe)	It can be useful for screening, but quantitation is hindered by poor databases of reference values.	

Wilkins et al. state that the following are examples of ideal sampling for the majority of medicinal and illegal drugs: antemortem samples (blood and urine); postmortem femoral/iliac venous blood; postmortem urine; vitreous (ideally fluoride oxalate preserved) [8].

Guidelines on Autopsy Practice on Individuals With Medical Devices

The following are Johnson et al.'s guidelines for pathologists performing postmortem investigations for patients who have implanted medical devices [6].

Risk assessment for the examination: The use of implanted defibrillators, which have the potential to shock anyone who comes into contact with them if they are not deactivated before a postmortem, has brought attention to potential risks for pathologists and other mortuary staff. Therefore, before beginning a postmortem, it is imperative to ensure that any such device is completely disabled [44]. Medical equipment can be divided into two major categories. The first category consists of the well-known intrauterine contraceptive devices and metal prosthetic devices used in joint surgery. Moreover, mesh devices are used to rebuild the abdomen and, occasionally, the perineum. Considering any signs of sepsis and evident sclerosis near these things is crucial [31]. According to Johnson et al., the second type of device is more interactive and includes traditional permanent pacemakers. However, defibrillator pacemakers are already widely used in medical therapy. Standard autopsy ought to reveal the existence of auditory loops, bladder stimulators (for spinal/cord sickness), and central nervous system stimulators for epilepsy.

Guidelines for pathologists who examine individuals who have implanted electronic medical devices after death [6]: An autopsy pathologist must consider the removal of bone-lengthening devices, such as fixation, before cremation. Penile pumps are among the strange equipment found in unusual places. Saukko et al. suggest that these can cause fatal occult sepsis [27].

Guidelines on Autopsy Practice: Procedures in Anesthesia-Related Deaths 

These are according to Helbert et al. and Attri et al. [5, 45]. In these cases, the main goal of the necropsy is to confirm or rule out natural sickness. It is difficult to investigate the pharmacological aspects of mortality connected to anesthesia. Studies on toxicology are usually useless, particularly when it comes to overdosing on drugs like barbiturates or adrenaline (epinephrine). Blood gas measurements cannot demonstrate hypoxia, and quantitative evaluations of volatile chemicals during necropsy are unreliable. When malignant hyperpyrexia is suspected antemortem blood samples must be drawn and examined.

The patient might have had several anesthetic devices during treatment, such as electrodes, catheters, cannulas, and endotracheal tubes. After death, things must stay where they are to guarantee correct placement and patency. Pre-necropsy imaging can assist in determining accurate anatomical positioning in cases when clinical data casts doubt on the location. Although esophageal intubation is uncommon, a primary midline neck incision can confirm it. A ring of edematous esophageal mucosa may be visible close to the inflated component if the position was adjusted after death.

Distention of the stomach and intestines can be caused by anesthetic gas. However, it is usually impossible to determine the exact source. An extensive histological investigation is advised in order to rule out occult intercurrent disease and gauge the extent and severity of the condition that needs to be surgically removed. Certain anesthetic effects, such as halothane hepatitis, can also be verified by histology. For neuropathological examination, keeping and analyzing the intact brain is essential, yet many deaths happen before hypoxia-related changes manifest.

Local and epidural anesthesia: Pöpping et al. documented that local anesthetics rarely cause death. The main risks include hypersensitivity and escape of adrenergic medications, which can lead to overdose. Diffusion from the injection site can result in vasoconstriction and cardiac collapse. Anesthetic concentrations in CSF fluid from necropsies should be examined. To examine the placement and patency of epidural catheters, a dye must be used and be carefully dissected [46].

Malignant hyperthermia: The diagnosis of malignant hyperthermia is based on clinical evidence, as there are no notable necropsy findings. Antemortem blood testing can verify the increased creatine phosphokinase and aldolase activity in 70% of carriers. It is advisable to test other family members for the condition. Certain anesthetic medications may present challenges when administered. Some anesthetic drugs can cause difficulties during administration. Repeated use of halothane anesthesia might lead to hepatitis. Excessive use of barbiturates during induction, including thiopentone (thiopental), can cause cardiac arrest [47, 48].

Autopsy report and clinicopathological summary

These are as stated in the Guidelines on Autopsy Practice: Postoperative Fatalities (May 2019) and ACP Best Practice no. 155: Pathological Investigation of Deaths Following Surgery, Anesthesia, and Medical Procedures (2000) [49,5]. Guidelines for autopsy reports have been established, including standards for substance and timeliness. Individual cases may require specific reports based on the surgical treatment performed. All reports should include three parts of information [50] which are as follows: actual findings of the autopsy; a brief explanation of the cause of death given; take into account if the death happened because of or despite the surgery; consider if the autopsy indicates any surgical or anesthetic error indications; if the cause of death and pathology are unclear, seek assistance from a more competent pathologist.

Explanation of These Findings and Conclusions

The pathological sequence should be logically laid forth in the final autopsy report and the clinicopathological summary. Clinicians, family members, solicitors, and members of the public and press will all closely examine the autopsy report. With input from surgeons, anesthesiologists, and other clinicians, a multidisciplinary approach may help the pathologist determine the most plausible and reasonable cause of death. In certain perioperative and postoperative deaths, the cause of death may not always be ascertained by the autopsy alone. One area fraught with difficulty is the declaration of the "cause of death." The British format is a recognized format that provides the reason for death. It indicates what caused the death; whether it was a disease or illness that caused death directly; other diseases or conditions, if any, leading to death; significant conditions that were not associated with the disease or condition that caused the death but contributed to its outcome.

Uncertain parts should be written in the opinions section, whereas genuine elements can be stated in the cause of death. If the cause of death cannot be identified, phrases like "undetermined" or "multiple factors" can be used. The ultimate phrasing is up to the competent medico-legal authorities.

Many pathologists have difficulty pinpointing the cause of death in postsurgical fatalities. The main question is whether the death would have happened without the procedure. In some circumstances, this may be impossible to identify. Any doubt should be clearly stated in the necropsy report. It might be challenging to determine the role of trauma and surgery in a death. When a patient dies from a sickness not related to the operation, it is important to distinguish between known and unknown disorders. When the disease is present, examine whether the operation was justified.

Unknown illnesses are only a concern if appropriate measures are not taken to identify common risk factors. In rare cases, a surgical or anesthetic procedure failure could be fatal. This could have been an accident, an inadvertent consequence of an extraordinarily challenging operation, anomalous anatomy, or malfunctioning equipment. Legal action for negligence may be taken if there is any possibility of individual error or incompetence and the pathologist must take great care to provide a thorough, unbiased, and objective autopsy report.

The autopsy report must meet the demands of several recipients, including medico-legal authorities, clinicians, family doctors, relatives, hospital management, medical insurers, and lawyers representing multiple parties. While it is hard to produce a report with a vocabulary everyone can understand, writing as simply as possible to convey technical information is important. The local coroner or equivalent medico-legal authority controls access to autopsy reports, which is contentious.

Local norms are crucial for conducting necropsies during surgical operations and anesthesia, which can pose confidentiality and disclosure challenges. Pathologists and doctors require adequate medico-legal training to collaborate with local coroners effectively.

Examples of cause of death opinions or assertions

Since surgery is a contributing factor to death, it needs to be taken into account when calculating the cause of death. It is not always a sign of error when surgical treatments are included in the cause-of-death determination (Table 5).

**Table 5:** Common causes of death formulation contributed to death after surgical interventions Source: [9]

System	Common causes of death	Common pathologies that are less often the direct cause of death
Cardiac	Cardiac tamponade, right ventricle rupture by temporary pacing wire, and complete heart block following myocardial infarct	Ischemic heart disease, hypertensive heart disease, cardiac air embolus, and central venous catheter placement
Respiratory	Pulmonary thromboembolism, hemothorax, laceration of the right subclavian vein, thoracoscopic pulmonary lobectomy for adenocarcinoma of the lung, developmental respiratory diseases, neoplasms of the respiratory system, pulmonary heart disease or diseases of pulmonary circulation, sleep-related breathing disorders, and diseases of the respiratory system complicating pregnancy, childbirth, or the puerperium	
Gastrointestinal	Anastomotic breakdown, particularly bowel fecal peritonitis, ischaemic anastomotic dehiscence, and sigmoid colectomy for adenocarcinoma of the sigmoid colon	Chronic obstructive pulmonary disease exacerbation following abdominal surgery and reduced mobility following right hemicolectomy for adenocarcinoma of the ascending colon
Bone	Bone cement implantation syndrome, cemented right hip hemiarthroplasty, and osteoporotic fracture of the right femoral neck	Reduced mobility and osteoporotic fracture of the left femoral neck (operated)
Sepsis	Bacterial sepsis (including pneumonia, urinary tract infection, wound infection, and peritonitis), and *Escherichia coli* septicemia	*Escherichia coli*-infected sacral pressure sores
Miscellaneous	Hemorrhage, potentially at any site	Intracranial hemorrhage, malignant hyperpyrexia, halothane hepatitis, drug-induced hepatitis (e.g., antibiotic-induced), and anaphylaxis

Medico-legal aspects of perioperative deaths

When a patient passes away during a surgical treatment carried out under anesthesia, the surgeon or anesthesiologist is frequently falsely blamed for the death. When a patient passes away while undergoing surgery while sedated, the anesthesiologist or surgeon should notify the police right away so that an inquest can be held. All fatalities after surgery and anesthesia should be considered unnatural and reported to the police. During the trial, the chief judicial officer may evaluate the following questions [15]: 1. Anesthesiologists are responsible for attending patients the day before surgery, doing a pre-anesthetic check-up, and conducting investigations. Before consent, the anesthesiologist should fully explain the operation, anesthetic drug, side effects, complications, and hazards to the patient; 2. Informed written consent: Before administering anesthesia, anesthesiologists must get written consent; 3. Anesthesiologists must use reasonable expertise and care while selecting anesthetic agents and performing procedures. Anesthesiologists must conform to conventional practices and institutional protocols.

Anesthesiologists may be liable for negligence if their actions or omissions result in patient harm, sickness, or death. Anesthesiologists can be held liable for negligence if injuries were caused solely by deviations from normal protocols during anesthesia operations. The plaintiff has the burden of showing the anesthesiologist's negligence. The court permits both parties to show their case through evidence. This could be records, books, journals, or expert testimonies. Carelessness is considered res ipsa loquitur when it is clear to a layperson; for instance, if a pre-anesthetic examination is not performed before administering anesthesia or if an inexplicable cardiac arrest during anesthesia results in death, this is considered carelessness. An explosion happened during the administration of anesthesia to a patient, despite the procedure being often employed without incident [51].

The defendant physician's responsibility, not the plaintiff's, is to demonstrate that their carelessness did not cause the accident. The court determined that the anesthetic agent was not contaminated and that the staff had taken the required procedures to disinfect themselves before the operation in a case where a patient acquired meningitis following spinal anesthesia. The hospital was fined for a sterilization process error. However, the anesthesiologist was not guilty [15].

In a notable medical malpractice case involving spinal anesthesia, the court highlighted the legal principle that it is the defendant physician's responsibility to demonstrate that their actions did not contribute to the adverse outcome, rather than the plaintiff's obligation to prove causation. In this instance, the patient developed meningitis postoperatively. The court found that the anesthetic agent used was uncontaminated and that the medical staff had adhered to all required disinfection protocols prior to the procedure. Despite the unfortunate outcome for the patient, the anesthesiologist was not found to be negligent. However, the hospital was penalized due to a failure in ensuring proper sterilization processes. This case underscores the complexities involved in medical malpractice litigation and the essential role of demonstrating adherence to medical standards of care [52].

Precaution and defense

Surgeons and anesthesiologists should maintain current, comprehensive patient records in addition to maintaining ongoing professional development. Before using any equipment, he/she must inspect it, check for drugs known to induce allergic responses and stay with the patient until they have fully recovered from the anesthetic's effects. An anesthesiologist can defend himself/herself against a negligence lawsuit by demonstrating that he/she used a fair amount of skill and care when performing anesthetic operations. A doctor is not negligent if he/she follows a practice approved by a responsible organization of medical professionals with training in that field prevalent at that time, even if other doctors may take a different course of action. This is Bolam's Law [53]. He/she is not liable as long as the physician exercised reasonable competence and care. The patient may also suffer harm due to therapeutic error, medical mishap, unanticipated injury, or the emergence of a new disease [54].

By helping to investigate the causes of perioperative accidents, one can streamline the auditing process, manage different clinical reactions, and determine whether doing so can alter unfavorable outcomes in the medico-legal field of interest. This theory states that the methods for patient safety that Madea et al. mentioned may assist in implementing in anesthetic-surgical practice will be revealed by a thorough multidisciplinary investigation that incorporates medico-legal data from autopsies, such as gross findings, additional laboratory analyses, and a recent molecular autopsy that revealed diseases that genes may prevent [55].

For the defense of anesthesiologists, Lee et al. recommended standard-of-care issues that indicated whether or not guidelines are applied are used to make choices about medical negligence, all within the framework of a medico-legal strategy [56].

Defensive medicine (DM)

Sadly, DM is gradually but steadily making its way into Egypt. Medical choices and activities regarding patient treatment should always be directed toward improving the patient's health. Whether it is an inquiry, a recommendation, a referral, or a procedure, the patient's best interests should always come first and foremost. This also holds for any guidance or instruction. However, even in these perfect circumstances, physicians occasionally find themselves in front of the courts, charged with malpractice for allegedly failing to take sufficient care of their patients. As medical malpractice claims against physicians became more likely than not, many doctors decided it was appropriate to incorporate certain measures into patient care that were not intended to improve the patient's health but rather to safeguard themselves in a malpractice lawsuit [57].

Multiple studies have highlighted how lawsuits negatively impact physicians, causing them stress and thereby threatening their future performance, according to Frati et al. [58]. Furthermore, "significant pressure on health professionals, particularly in some specialized branches more exposed to this risk," is also due to it. De Ville et al. state that "physicians are likely to look to the law first, not afterward, and are often preoccupied with maintaining the safest legal procedure possible" [59]. On the other hand, Frati et al. emphasize that "the idea that a fear of lawsuits can lower the rate of medical errors is not supported by the literature" [58].

According to Tuers et al., DM comes in two main forms. In an active form, which is often referred to as "positive," the doctor will request additional tests and procedures.". In terms of avoiding high-risk patients, the other is "passive or negative." Defensive actions can also be divided into two main categories: "avoidance practices, which try to keep away from the treatment of the high patients, and assurance practices, which needlessly overinvestigate the low-risk patients" [60].

Baungaart et al. point out that fear of litigation is not the only existing form. These other forms, motivated by "self-protective motives," fall into four categories: fear of patient dissatisfaction, fear of failing to recognize a serious diagnosis, fear of bad press, and unconscious defensive medicine [61]. According to this viewpoint, doctors who face legal action are more cautious in their activities and protocols to avoid medical claims "rather than to promote the patient's best interest," disobeying medical ethics [62].

These additional kinds, driven by "self-protective motives," can be broadly classified into four categories: fear of negative press, worry of failing to recognize a dangerous diagnosis, fear of patient dissatisfaction, and unconscious defensive medicine. This argument holds that 21 physicians under legal threat behave more cautiously and follow procedures "rather than to promote the patient's best interest," which is against medical ethics [63].

The fact that DM is neither harmless nor innocuous is another issue. Due to the likelihood of lawsuits against surgeons performing preoperative or surgical treatments, requests for unnecessary tests before procedures can improve the whole process [63].

Recommendations

A comprehensive multidisciplinary investigation that includes medico-legal data from autopsies, such as gross findings, additional laboratory analyses, and a recent molecular autopsy that revealed diseases that genes may prevent, will reveal the patient's safety strategies. In addition, standard-of-care issues that show whether or not guidelines are applied are utilized to make decisions concerning medical negligence, all within the framework of a medico-legal strategy.

Careful analysis and inquiry are needed for deaths that result from medical procedures and complications. The cause of death sequence should state whether the operation was a factor in the death. Every report should include a clinicopathological correlation that evaluates the case and the reliability of the findings drawn from the data.

Local guidelines and nationally consistent standards and criteria should be in place to investigate reported fatalities. This covers the diagnostic stage of the inquiry, as autopsies related to surgery and anesthesia would most likely raise delicate questions about privacy and transparency.

Independent peers should regularly review autopsy reports and procedures to ensure uniformity of established standards and accountability. In addition, pathologists and coroners should review each other's autopsy reports and related documents while undergoing training and as part of their ongoing professional development.

It is important to create additional perioperative quality control initiatives and examine their applicability to medico-legal assessments.

## Conclusions

Among the most taxing events experienced by surgeons and anesthesiologists is death associated with surgery and anesthesia. Surgeons and anesthesiologists are caught at least once in this tragedy. Many pathologists have problems determining the cause of perioperative deaths. Their main role is to determine if death occurred due to a disease other than the procedure for which it was performed. A multidisciplinary approach with input from surgeons, anesthesiologists, and other clinicians may help the pathologist establish the most likely or reasonable cause of death. An autopsy alone may not be able to determine the cause of death in all perioperative deaths.

In order to develop a strategy for averting possibly deadly complications, forensic pathology professionals need to take into account such fatal events in terms of malpractice claims. So, efforts are being made to release guidelines for forensic pathologists to follow in order not to miss any leading information that could help in detecting the cause of death. Local rules are crucial since autopsies related to surgery and anesthesia will always bring up challenging questions about disclosure and secrecy. Errors in judgment and performance occur and can have serious consequences.
